# P-781. The Impact of Hepatitis B Virus Infection on the Management of Latent Tuberculosis Infection in Northern California, 2010-2022

**DOI:** 10.1093/ofid/ofae631.975

**Published:** 2025-01-29

**Authors:** Julia R Fink, Zinnia Dong, Matthew Murrill, Robert J Wong, Amit S Chitnis, Devan Jaganath, Amy S Tang

**Affiliations:** NYU Langone Health, New York, New York; North East Medical Services, San Francisco, California; University of California, San Francisco, San Francisco, California; Stanford Medicine, Stanford, California; Alameda County Public Health Department, San Leandro, California; University of California, San Francisco, San Francisco, California; North East Medical Services, San Francisco, California

## Abstract

**Background:**

Tuberculosis (TB) and hepatitis B virus (HBV) infection affect similar at-risk populations. However, there is limited data on how co-infection may influence clinicians’ decision to offer TB preventative treatment (TPT) over concern for hepatotoxicity. We compared TPT by HBV status, and determined factors associated with TPT initiation among HBV-infected persons at a community health center in Northern California serving a large non-U.S.-born Asian population.

**Figure:**
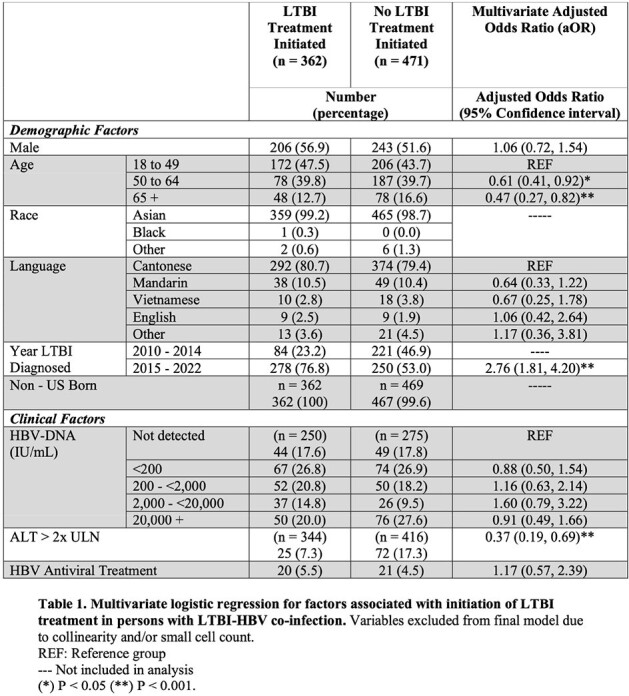

**Methods:**

We extracted electronic health record data for all persons aged ≥18 years old with latent TB infection (LTBI), defined by a positive tuberculin skin test or interferon-gamma release assay in the absence of a TB disease diagnosis from 2010-2022. HBV infection was defined by a single positive HBV DNA or HBV surface antigen assay. We conducted multivariate logistic regression to explore the impact of (a) HBV status and (b) clinical and demographic variables on TPT initiation. We also examined how these factors changed when Rifampin (RIF) usage for TPT increased at this community health center after 2014.

**Results:**

A total of 11,711 persons had LTBI and 5,530 had HBV. Of all persons with LTBI, 833 (9.0%) of those tested for HBV had a co-infection including 41 (4.9%) on HBV antiviral treatment; while 34% of all HBV-infected persons screened for TB had LTBI. Adjusting for demographics and time period, TPT was significantly lower among those with co-infection than those without (43.5% versus 47.4%, adjusted Odds Ratio (aOR) 0.76, 95% Confidence Interval (CI) 0.66 – 0.88, p< 0.01). Among those with co-infection, increasing age (50-64, ≥65 years), and elevated alanine transaminase (ALT) levels were associated with decreased likelihood of TPT (Table 1). Treatment of co-infected individuals was significantly higher after 2014 when RIF prescribing increased (aOR 2.76, 95% CI 1.81-4.2); however, age over 65 was still associated with decreased TPT prescription (aOR 0.36, 95% 0.19 – 0.67, p < 0.01).

**Conclusion:**

HBV-LTBI co-infection was associated with reduced odds of TPT. Increased use of Rifamycin based regimens and further guidance on TPT and monitoring of older individuals and persons with elevated ALT levels may be needed to improve TB prevention efforts among HBV-LTBI co-infected persons.

**Disclosures:**

**Robert J. Wong, MD, MS**, Durect Corporation: Grant/Research Support|Exact Sciences: Grant/Research Support|Gilead Sciences: Grant/Research Support|Thera Technologies: Grant/Research Support

